# The D405N Mutation in the Spike Protein of SARS-CoV-2 Omicron BA.5 Inhibits Spike/Integrins Interaction and Viral Infection of Human Lung Microvascular Endothelial Cells

**DOI:** 10.3390/v15020332

**Published:** 2023-01-24

**Authors:** Antonella Bugatti, Federica Filippini, Serena Messali, Marta Giovanetti, Cosetta Ravelli, Alberto Zani, Massimo Ciccozzi, Arnaldo Caruso, Francesca Caccuri

**Affiliations:** 1 Section of Microbiology, Department of Molecular and Translational Medicine, University of Brescia, 25123 Brescia, Italy; 2 Laboratório de Flavivirus, Instituto Oswaldo Cruz, Rio de Janeiro 21040-900, Brazil; 3 Department of Science and Technology for Humans and the Environment, University Campus Bio-Medico of Rome, 00128 Rome, Italy; 4 Section of General Pathology, Department of Molecular and Translational Medicine, University of Brescia, 25123 Brescia, Italy; 5 Unit of Medical Statistics and Molecular Epidemiology, University Campus Bio-Medico of Rome, 00128 Rome, Italy; 6 Institute of Human Virology, Department of Medicine, University of Maryland School of Medicine, Baltimore, MD 21201, USA

**Keywords:** SARS-CoV-2, Omicron variant, endothelial cell dysfunction, integrins

## Abstract

Severe COVID-19 is characterized by angiogenic features, such as intussusceptive angiogenesis, endothelialitis, and activation of procoagulant pathways. This pathological state can be ascribed to a direct SARS-CoV-2 infection of human lung ECs. Recently, we showed the capability of SARS-CoV-2 to infect ACE2-negative primary human lung microvascular endothelial cells (HL-mECs). This occurred through the interaction of an Arg-Gly-Asp (RGD) motif, endowed on the Spike protein at position 403–405, with α_v_β_3_ integrin expressed on HL-mECs. HL-mEC infection promoted the remodeling of cells toward a pro-inflammatory and pro-angiogenic phenotype. The RGD motif is distinctive of SARS-CoV-2 Spike proteins up to the Omicron BA.1 subvariant. Suddenly, a dominant D405N mutation was expressed on the Spike of the most recently emerged Omicron BA.2, BA.4, and BA.5 subvariants. Here we demonstrate that the D405N mutation inhibits Omicron BA.5 infection of HL-mECs and their dysfunction because of the lack of Spike/integrins interaction. The key role of ECs in SARS-CoV-2 pathogenesis has been definitively proven. Evidence of mutations retrieving the capability of SARS-CoV-2 to infect HL-mECs highlights a new scenario for patients infected with the newly emerged SARS-CoV-2 Omicron subvariants, suggesting that they may display less severe disease manifestations than those observed with previous variants.

## 1. Introduction

SARS-CoV-2 is the causal agent of the new pandemic disease COVID-19. The spectrum of clinical manifestations following SARS-CoV-2 infection spans from very mild to severe symptoms [1], while asymptomatic cases of this disease further its infectious spread [2,3]. When SARS-CoV-2 infection progresses to severe disease, patients may develop acute respiratory distress syndrome (ARDS). Symptoms and signs of severely infected patients are comparable to those shown by patients suffering from classic ARDS and exhibit the same pathophysiological mechanisms [4,5], mainly reflecting a dramatic human lung microvascular endothelial cell (HL-mECs) dysfunction [6]. This is characterized by neovessel formation through a mechanism of intussusceptive angiogenesis [7] and inflammation of the endothelium (endothelialitis), with changes in vascular permeability [4,8,9,10] and activation of procoagulant pathways [11]. Pathological evaluation of lung autopsies has uncovered evidence of viral particles associated with HL-mECs [7,12]. This indicates that distinct angiocentric features of severe COVID-19 may be sustained by a direct SARS-CoV-2 infection of HL-mECs. SARS-CoV-2 utilizes the host angiotensin-converting enzyme 2 (ACE2) as a target for penetration, fusion, and replication into a host cell [13,14,15]. However, glycan binding sites of the virus’s Spike protein are key to the initial binding of the virus to host cells [16,17,18,19]. In a normal adult human lung, ACE2 is expressed primarily in alveolar epithelial type II cells, whereas it is not expressed in HL-mECs [20]. We recently showed that SARS-CoV-2 is able to infect ACE2-negative HL-mECs by using an endocytic pathway [21]. Following its entry, SARS-CoV-2 promotes a potent pro-angiogenic and inflammatory microenvironment without releasing virions and in the absence of any cytopathic effect. This finding led us to investigate new possible receptors for SARS-CoV-2 on HL-mECs. A unique Arg-Gly-Asp (RGD) motif is expressed in the Receptor Binding Domain (RBD) at amino acid positions 403 to 405, outside the ACE2 recognition site [20]. This RGD motif is known to be responsible for binding to different integrins, thus introducing their potential role as receptors for SARS-CoV-2 entry into target cells [22,23]. Interestingly, the RGD motif is peculiar to SARS-CoV-2, being it is not expressed on the Spike protein of any other known human or animal coronaviruses [20]. In a recent study, we showed that the RGD motif is responsible for SARS-CoV-2 entry into HL-mECs through a specific α_v_β_3_ integrin interaction, giving rise to the release of a plethora of pro-inflammatory and pro-angiogenic molecules [20,21]. Proteome analysis confirmed a remodeling of SARS-CoV-2-infected HL-mECs to inflammatory and angiogenic responses. Inhibition of the Spike RGD/integrins interaction resulted in blocking SARS-CoV-2 infection and in a complete recovery of viral-induced HL-mEC dysfunction [20]. Our data highlighted the RGD motif as a functional constraint aimed at maintaining the interaction of the Spike protein with integrins. Omicron BA.2, BA.4, and BA.5 differ from all previous SARS-CoV-2 variants of concern (VOCs) and display a dominant D405N mutation in their Spike protein. A recent study showed that the D405N mutation has emerged in the Omicron sublineage BA.2 to evade humoral immunity elicited by Omicron BA.1 infection [24]. Here we show that the D405N mutation on the Spike of Omicron BA.5 inhibits SARS-CoV-2 capability to interact with integrins and, consequently, the inability of an Omicron BA.5 primary isolate to infect HL-mECs, thereby providing protection against virus-induced EC dysfunction. This dramatic loss of function attests to an evolutionary trajectory of SARS-CoV-2-emerging variants in hostile human environments that affect virus biology and pathogenesis to preserve transmission fitness.

## 2. Materials and Methods

### 2.1. Cells

African green monkey kidney Vero E6 cell line was obtained from Istituto Zooprofilattico Sperimentale della Lombardia e dell’Emilia Romagna (Brescia, Italy) and maintained in Dulbecco’s Modified Eagle Medium (DMEM; Gibco, Thermo Fisher Scientific, Waltham, MA, USA) supplemented with 10% fetal bovine serum (FBS; Gibco, Thermo Fisher Scientific). HL-mECs were purchased from Lonza Clonetics (Walkersville, MD, USA) and cultured in EGM-2 MV (Lonza, Basel, Switzerland) containing 10% FBS.

### 2.2. Monitoring of SARS-CoV-2 Emerging Variant and Phylogenetic Analysis

The genomic sequence dataset evaluated in the current study was based on 6,910,627 million full-length SARS-CoV-2 sequences, available in the Global initiative on sharing all influenza data (GISAID) until October 2022 [25,26]. Globally, the trend of surging D405N mutation across VOCs sequence was customly evaluated, excluding low-quality genomes. Phylogenetic analysis on SARS-CoV-2 genomic data was conducted using a set of representative data (*n* = 731) collected up to November 2022, belonging to different lineages (Pre-Omicron, B.1.1.529, Omicron BA.1, the few available strains belonging to the Omicron BA.1 lineage which showed the D405N mutation, Omicron BA.2, Omicron BA.4 and Omicron BA.5) [27]. All sequences were aligned using the ViralMSA tool [28,29], and IQ-TREE2 [30] was used for phylogenetic analysis following the maximum likelihood (ML) approach. TreeTime [31] was used to transform this ML tree topology into a dated tree using a constant mean rate of 8.0 × 10^−4^ nucleotide substitutions per site per year after the exclusion of outlier sequences.

### 2.3. Viral Infection

Infections were carried out as previously described [32], using the clinical SARS-CoV-2 isolates belonging to Omicron BA.1 (GISAID accession number: EPI_ISL_15700833) or Omicron BA.5 (GISAID accession number: EPI_ISL_15082179) sublineages. The viruses were propagated in Vero E6 cells, and the viral titer was determined by a standard plaque assay. All the experiments were performed with a single viral inoculum. Mock-infected cell cultures were obtained from uninfected cells and processed exactly as the SARS-CoV-2-infected ones. All the infection experiments were carried out in a biosafety level-3 (BSL-3) laboratory at a Multiplicity of Infection (MOI) of 1.

### 2.4. Surface Plasmon Resonance (SPR)

SPR measurements were conducted on a Biacore X100 (Cytiva, Washington, DC, USA) at 25 °C in order to characterize the interaction of the Omicron BA.1 and BA.5 sublineages’ receptor binding domain (RBD) with the α_v_β_3_ integrin immobilized on a CM5 sensor chip. To this aim, 20 µg/mL of α_v_β_3_ integrin (R&D systems, Minneapolis, MN, USA) were loaded onto the chip allowing the immobilization of 292 resonance units (RU), equal to 1.62 fmol/mm^2^ of integrin. An empty sensor chip was used to evaluate nonspecific binding and for blank subtraction [33]. Recombinant RBD of Omicron BA.1 or Omicron BA.5 (R&D systems) at a concentration of 1500 nM was diluted in 10 mM Hepes pH 7.4 containing 0.15 mM NaCl, 50 µM EDTA, 0.005% Surfactant P20, 1 mM CaCl_2_, 1 mM MgCl_2_ and 1 mM MnCl_2_ (running buffer) and injected over integrin or blank surfaces for 2 min and then washed until dissociation. After each run, the sensor chip was regenerated by injection of 2 M NaCl. Since its binding to integrin, increasing concentrations (from 125 to 1500 nM) of the Omicron BA.1 RBD were used to determine the kinetic parameters of its interaction with immobilized α_v_β_3_ integrin. The affinity values were calculated from the sensorgram overlays by using the nonlinear fitting single-site model software package BIAevaluation (version 3.2 [Cytiva]). Only sensorgrams whose fitting gave χ^2^ values close to 10 were used [34]. 

### 2.5. Viral RNA Extraction and qRT-PCR

RNA was extracted from infected cells using RNeasy Plus mini kit (Qiagen, Hilden, Germany), according to the manufacturer’s instructions. RNA was eluted in 30 μL of RNase-free water and stored at −80 °C until use. The qRT-PCR was carried out following previously described procedures [35]. Briefly, reverse transcription and amplification of the S gene were performed using the one-step QuantiFast Sybr Green RT-PCR mix (Qiagen) as follows: 50 °C for 10 min; 95 °C for 5 min; 95 °C for 10 s; 60 °C for 30 s (40 cycles) (primers: RBD-qF1: 5′-CAA TGG TTT AAC AGG CAC AGG-3′ and RBD-qR1: 5′-CTC AAG TGT CTG TGG ATC ACG-3′). A standard curve was obtained by cloning the receptor binding domain of the S gene (primers: RBD-F: 5′-GCT GGA TCC CCT AAT ATT ACA AAC TTG TGC C-3′; RBD-R: 5′-TGC CTC GAG CTC AAG TGT CTG TGG ATCAC-3′) into pGEM T-easy vector (Promega, Madison, WI, USA). A standard curve was generated by the determination of copy numbers derived from serial dilutions (10^3^–10^9^ copies) of the plasmid. Each quantification was run in triplicates. The 2^−DDCq^ (Livak) method was used for the comparison of the target qPCR product with the standard curve.

### 2.6. Immunofluorescence Assay and Microscopy Analysis

HL-mECs were seeded (5 × 10^4^ cells per well) on collagen-coated 8-well chamber slides (Thermo Fisher Scientific) and infected with SARS-CoV-2, belonging to the Omicron BA.1 and BA.5 sublineages, as described above. Twenty-four h post-infection (p.i.), cells were fixed with 4% paraformaldehyde (PFA) in Phosphate-buffered saline (PBS) for 10 min, permeabilized with 0.1% Triton X-100 in PBS, and saturated with 3% Bovine serum albumin ((BSA) Merck KGaA, Darmstadt, Germany), 0.1% Tween 20 in PBS. For staining, cells were incubated for 1 h with a human serum containing IgG to SARS-CoV-2 (collected from volunteers who received the BNT162b2 vaccine, 1:1000 dilution) and anti-GM130 antibody as a cis-Golgi marker (Abcam, Cambridge, UK), followed by Alexa Fluor 488-conjugated anti-human IgG or Alexa Fluor 647-conjugated anti-rabbit IgG (Thermo Fisher Scientific). Nuclei were counterstained with 4′,6-diamidino,2-phenylindole ((DAPI), Merck KGaA). Mock cells were used as a negative control. Fluorescence was recorded using a Zeiss Axiovert 200 epifluorescence microscope equipped with a Plan-Neofluar 20×/0.5 NA and a Plan-Apochromat 63×/1.4 NA objectives and with an Apotome imaging system (Zeiss Axiovert 200M system). Image quantification was performed using Fiji image analysis software (NIH, Bethesda, USA). Briefly, the Spike-related fluorescent area was quantified in 20–30 Omicron BA.1 and Omicron BA.5 infected cells and subtracted from the non-infected cells’ fluorescence signal. The decrease of Spike-related fluorescence in Omicron BA.5 cells was related to the Omicron BA.1 signal.

### 2.7. Microarray Analysis

Supernatants from infected HL-mECs were collected at 3 days p.i., clarified, and analyzed for the expression of 55 different angiogenesis-related proteins by the Human Angiogenesis Array Kit (Proteome Profiler, R&D systems) or for the expression of 36 different cytokine-related proteins by the Human Cytokine Array Kit (Proteome Profiler, R&D systems) according to the manufacturer’s instructions.

### 2.8. Tube Formation Assay

Tube formation assays were performed as previously described, with minor modifications [36]. Briefly, 150 μL of Cultrex Reduced Growth Factor Basement Membrane Extract (RGF BME) (Trevigen Inc., Gaithersburg, MD, USA) were transferred to prechilled 48-well culture plates. Plates were incubated for 1 h at 37 °C. Cells were collected at day 3 p.i., resuspended in the culture medium containing 10% FBS, seeded 4.5 × 10^4^ per well, and analyzed for tube formation at 12 h after cell seeding by examination with a Leica DM IRB microscope (Leica, Wetzlar, Germany). The center of each well was digitally photographed with a Hitachi KP-D50 camera (Hitachi, Tokyo, Japan), and capillary-like structures were quantified by analyzing the number of tubes per well formed by ECs. 

### 2.9. Co-Cultivation

Co-culture between infected and non-infected HL-mECs was performed in the absence of direct cell contact by using transwell inserts (polycarbonate filters coated with collagen, 0.4 μm pore size, Corning, New York, NJ, USA). Briefly, HL-mECs seeded in the lower compartment were infected as described above, while non-infected HL-mECs were seeded on the collagen-coated inserts in the upper chamber. After 3 days, cells in the upper well were trypsinized and used to perform the tube formation assay.

### 2.10. Statistical Analysis

Data were analyzed for statistical significance using the Student’s t-test or one-way ANOVA when appropriate. The Bonferroni post-test was used to compare data. Differences were considered significant when *p* < 0.05. Statistical tests were performed using Prism 8 software (GraphPad Software, La Jolla, CA, USA).

## 3. Results

### 3.1. Mutations in the Integrin-Binding RGD Motif of SARS-CoV-2 Variants

**Figure 1:**
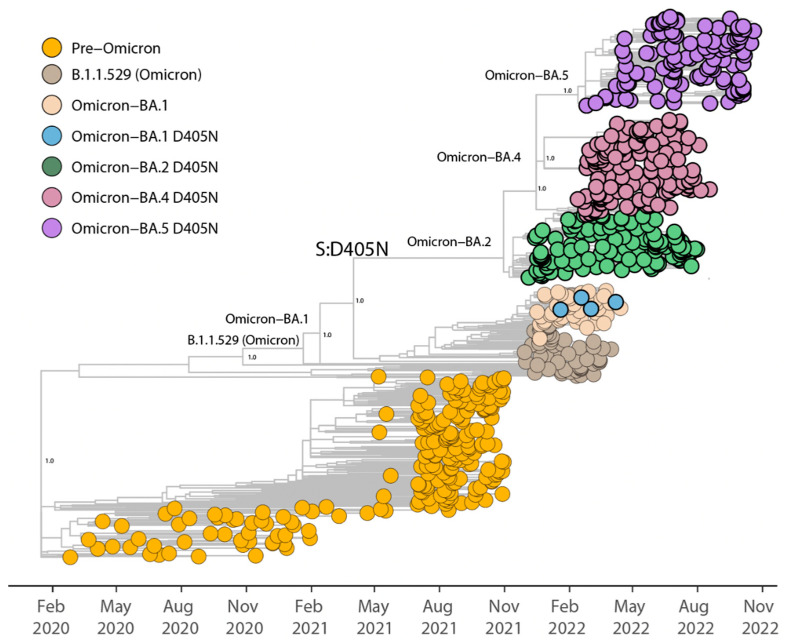
Time-resolved maximum-likelihood tree of SARS-CoV-2, including representative worldwide subsample genomes (*n* = 731) collected up to November 2022. The genomes are colored according to the lineages (VOC and ancestral lineages), as shown in the legend on the top left. Time of insurgence of the Spike (S):D405N mutation is highlighted in the figure. Statistical support (SH-aLTR = 1.0) is shown at key nodes.

We recently demonstrated that SARS-CoV-2 is capable of infecting human primary HL-mECs by using integrins as an alternate receptor to ACE2 [20,21]. SARS-CoV-2/integrins interaction occurs through a conserved RGD motif [amino acid (aa) 403–405] that is peculiar for SARS-CoV-2 Spike protein, while it is absent in all other known coronaviruses. To analyze the evolution of the RGD motif in SARS-CoV-2 variants, we evaluated 6,910,627 SARS-CoV-2 viral genomes available in the GISAID database from December 2019 to October 2022. Sequences were partitioned by pangolin lineage to identify emerging mutations in the RGD motif at the global level. We found that the RGD motif has been well conserved in all Pre-Omicron SARS-CoV-2 lineages. Indeed, only 0.02% of total sequences collected before the Omicron variant advent showed an aa change in positions 403, 404, or 405. As reported in Table 1, a very low percentage (0.01%) of total isolates showed mutations in the RGD motif at aa positions 403 and 404, and almost all these mutations occurred in the Spike of Pre-Omicron lineages. However, since the emergence of the first Omicron sublineage, different aa substitutions in position 405 started to appear, with 1164 mutated isolates found from a total of 422,746 BA.1 deposited sequences (0.28%). The fixation of the D405N mutation was estimated to have occurred in May 2021, with the emergence of the Omicron BA.2 lineage. From this moment, the percentage of SARS-CoV-2 isolates in the world carrying the D405N mutation has been stable at around 97–98% (Table 1). To better understand the time of emergence and the evolution of the D405N mutation, a set of 731 representative whole genome sequences were collected up to November 2022. They represented the sequence diversity at the time of analysis and were submitted to phylogenetic inference. As shown in Figure 1, by the end of April-beginning of May, the D405N mutation starts to become predominant in other previously wild-type lineages. Almost all sequences belonging to Omicron BA.2, BA.4, and BA.5, in addition to a few strains belonging to the BA.1 lineage, were found to carry this aa change in the RGD motif. This finding leads to the hypothesis of the emergence of D405N mutation as a result of viral evolution and adaptation aimed to enhance viral fitness in the human host.

### 3.2. D405N Mutation in the Spike of SARS-CoV-2 Omicron BA.5 Inhibits Spike/Integrins Interaction

**Figure 2:**
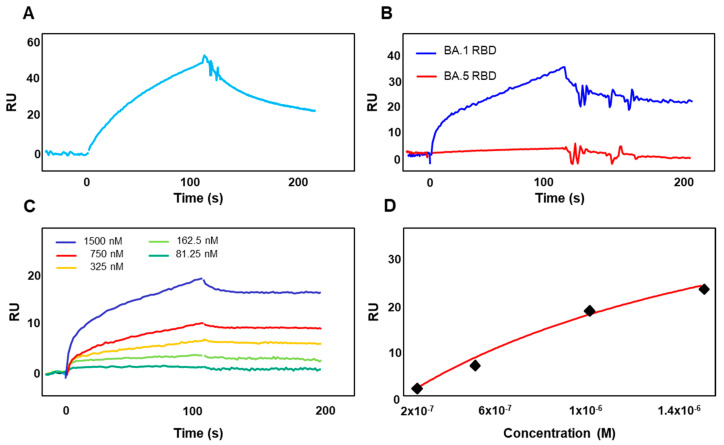
Recombinant RBD of SARS-CoV-2 Spike protein belonging to Omicron BA.5 does not bind to immobilized α_v_β_3_ integrin. (**A**) The specificity of the integrin surface was demonstrated by the binding of the monoclonal antibody against α_v_β_3_. (**B**) RBD of SARS-CoV-2 Spike protein belonging to Omicron BA.1 (blue line) or Omicron BA.5 (red line) sublineages at a concentration of 1500 nM was injected onto immobilized α_v_β_3_ integrin. (**C**) Sensogram overlay showing the binding of increasing amounts of the RBD of SARS-CoV-2 Spike protein belonging to Omicron BA.1 (from 81.25 to 1.500 nM) sublineage to immobilized α_v_β_3_ integrin. The response in resonance units (RU) was recorded as a function of time. (**D**) Saturation curve obtained by using the values of RU bound at equilibrium from the injection of increasing concentrations of free RBD onto sensorchip immobilized α_v_β_3_ integrin.

Our previous data highlighted the role played by the RGD motif in allowing SARS-CoV-2 entry into ACE2-negative HL-mECs through binding to α_v_β_3_ integrin expressed on the cell surface [20,21]. The evidence for a critical mutation in the integrin-binding RGD motif prompted us to study if the most recently developed sublineage, namely Omicron BA.5, which carries the same D405N mutation in the RGD motif as the Omicron BA.2 and BA.4 sublineages, was still able to use integrins as receptors for entry into target cells. To clarify this point, we performed a SPR study using the BIAcore technology. To this aim, α_v_β_3_ integrin was immobilized onto a Biacore CM5 sensor chip, and the demonstration of integrin integrity on the chip surface was obtained by binding a monoclonal antibody to α_v_β_3_ integrin (Figure 2A). As expected, the RBD of SARS-CoV-2 Spike protein belonging to Omicron BA.1 sublineage (1500 nM), and displaying the RGD motif, was able to bind to immobilized α_v_β_3_ integrin (Figure 2B, blue line). On the contrary, the RBD of SARS-CoV-2 Omicron BA.5 sublineage (1500 nM) expressing the D405N mutation completely lost its α_v_β_3_ integrins binding ability (Figure 2B, red line). The RBD of Omicron BA.1 sublineage was able to bind to immobilized α_v_β_3_ in a dose-dependent manner (Figure 2C) and with an affinity value equal to 4.6 nM (Figure 2D). This finding confirms previous results [21] and further attests to the specificity of the Omicron BA.1 RBD/α_v_β_3_ integrin interaction. 

### 3.3. Lack of SARS-CoV-2 Omicron BA.5/Integrin Interaction Impairs Virus Entry into HL-mECs

**Figure 3:**
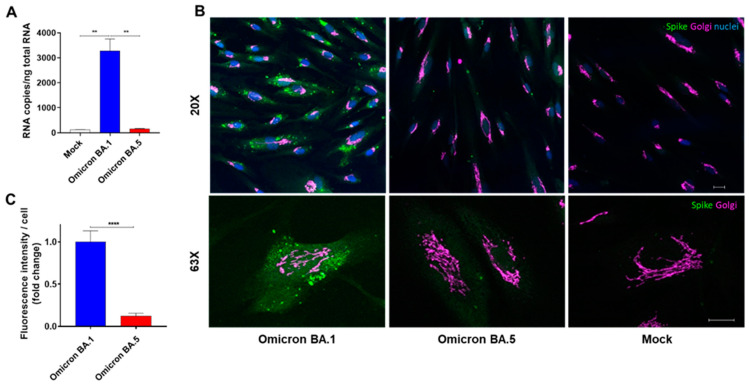
Omicron BA.5 does not infect HL-mECs. HL-mECs were mock-infected (Mock) or infected with Omicron SARS-CoV-2 belonging to BA.1 or BA.5 sublineages (Omicron BA.1 and Omicron BA.5, respectively) at an MOI of 1 for 1 h at 37 °C, then washed and cultured until day 1 p.i. (**A**) The graph shows the quantitation of SARS-CoV-2 genomes at the intracellular level by qRT-PCR. At least three replicates were performed. Data are representative of two independent experiments with similar results. Statistical analysis was performed by one-way ANOVA (** *p* < 0.01). (**B**) HL-mECs were fixed with 4% PFA in PBS, permeabilized with 0.1% Triton X-100 in PBS, and saturated with 0.1% BSA, 0.1% Tween 20 in PBS. For staining, cells were incubated for 1 h with a human serum containing IgG to SARS-CoV-2 and anti-GM130 antibody as cis-Golgi marker followed by Alexa Fluor 488-conjugated anti-human IgG or Alexa Fluor 647-conjugated anti-rabbit IgG. Nuclei were counterstained with DAPI (scale bar, 20 µm). Images display SARS-CoV-2 signals in green and cell nuclei in blue. (**C**) The Spike-related fluorescent area was quantified in 20–30 Omicron BA.1- and Omicron BA.5-infected cells and subtracted of mock cells fluorescence signal. The decrease of Spike-related fluorescence in Omicron BA.5 cells was related to Omicron BA.1 signal and was expressed as fold change. Statistical analysis was performed by unpaired *t*-test (**** *p* < 0.0001).

Recently, we showed that SARS-CoV-2 infects human ACE2-negative HL-mECs through an α_v_β_3_ integrin-mediated endocytosis [20]. Since the D405N mutation was found to inhibit Spike/α_v_β_3_ integrins interaction, we wondered whether this mutation could neutralize SARS-CoV-2 infection of HL-mECs. To test this hypothesis, cells were infected with 1 MOI of authentic SARS-CoV-2 Omicron BA.1 and BA.5 viruses. Twenty-four hours p.i., SARS-CoV-2 viral genome and protein expression were evaluated by quantitative real-time PCR and indirect immunofluorescence assay, respectively. As shown in Figure 3A, quantification of intracellular SARS-CoV-2 RNA showed the absence of viral RNA in SARS-CoV-2 Omicron BA.5-infected HL-mECs as compared to SARS-CoV-2 Omicron BA.1-infected cells. Similar results were obtained by evaluating viral protein expression in HL-mECs by immunofluorescence assay. As shown in Figure 3B, the presence of SARS-CoV-2 Spike protein was observed in Omicron BA.1-infected HL-mECs, whereas viral protein expression was not observed in the Omicron BA.5-infected ones. As expected, the presence of the viral protein was not detected in mock-infected cells used as a control. Quantification of the Spike-related fluorescence in Omicron BA.5-infected cells confirmed the absence of viral proteins (Figure 3C). Taken together, these findings demonstrate that, differently from the RGD-expressing Omicron BA.1 isolate, SARS-CoV-2 Omicron BA.5, carrying the D405N mutation in the integrin-binding motif, is unable to gain access into HL-mECs. 

### 3.4. SARS-CoV-2 Omicron BA.5-Infected HL-mECs Do Not Release Inflammatory Cytokines and Angiogenic Molecules

**Figure 4:**
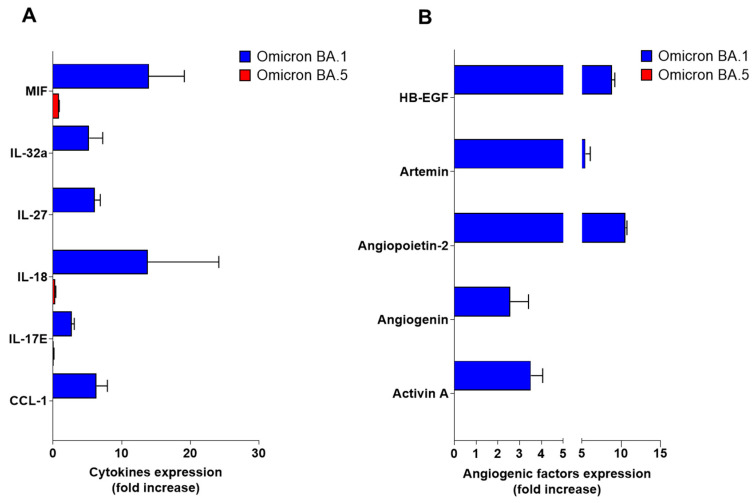
Release of inflammatory cytokines and angiogenic molecules from HL-mECs upon SARS-CoV-2 Omicron BA.1 or BA.5 infection. HL-mECs were mock-infected (Mock) or infected with SARS-CoV-2 belonging to Omicron BA.1 or BA.5 lineages (Omicron BA.1 and Omicron BA.5, respectively) at MOI 1 for 1 h at 37 °C and then washed and cultured until day 3 p.i. Then, supernatants were evaluated for the presence of (**A**) cytokines and (**B**) angiogenic molecules by human proteome arrays. The results are expressed as mean values ± SD of duplicates given as fold increase as compared to Mock cells. Data are representative of one out of two independent experiments with similar results.

In order to understand whether SARS-CoV-2 Omicron BA.5 inability to enter into HL-mECs was impacting cell functions, we analyzed the infected-HL-mECs secretome at day 3 p.i., by using human angiogenic and cytokine arrays. As shown in Figure 4A, Omicron BA.1-infected HL-mECs (blue bars) released different pro-inflammatory molecules, among which one of the most expressed was IL-18. It is worth noting that activation of the IL18/IL18R1/HIF-1 signaling axis was found to mediate an increased risk of peripheral vascular diseases such as aneurysms and atherosclerosis after COVID-19 [37]. On the other hand, Omicron BA.5-infected HL-mECs (red bars) did not secrete any pro-inflammatory cytokines. At the same time, Omicron BA.1-infected HL-mECs promoted the secretion of different angiogenic factors (Figure 4B). On the contrary, Omicron BA.5-infected HL-mECs did not release angiogenetic factors (Figure 4B).

### 3.5. SARS-CoV-2 BA.5 Does Not Trigger Angiogenesis

**Figure 5:**
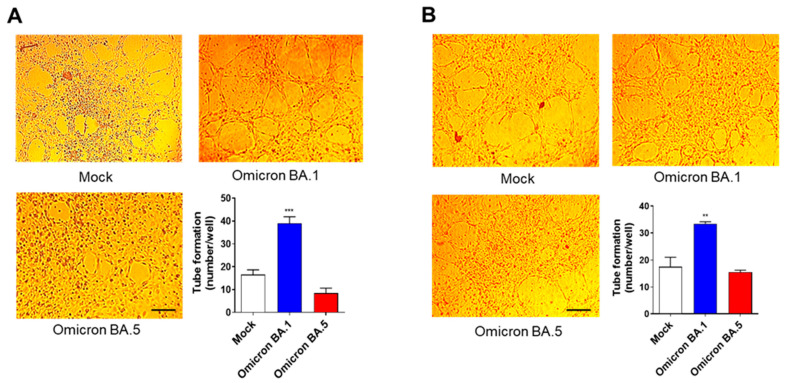
SARS-CoV-2 BA.5 does not trigger angiogenic functions. (**A**) HL-mECs were mock-infected (Mock) or infected with SARS-CoV-2 belonging to Omicron BA.1 or BA.5 lineages (Omicron BA.1 and Omicron BA.5, respectively) at MOI 1, for 1 h at 37 °C and then washed and cultured until day 3 p.i. Mock-, Omicron BA.1-, and Omicron BA.5-infected HL-mECs were seeded on reduced growth factor Matrigel-coated wells and then cultured for 12 h at 37 °C. Pictures are representative of one out of three independent experiments with similar results (scale bar, 200 µm). (**B**) Tube formation assay performed with HL-mECs co-cultivated for 3 days with Mock, Omicron BA.1, or Omicron BA.5-infected HL-mECs. The pictures were taken 12 h after cell seeding (scale bar, 200 µm). Pictures are representative of one out of three independent experiments with similar results. Values reported in the graphs are the mean ± SD of one representative experiment out of three independent experiments with similar results performed in triplicate. Statistical analysis was performed by one-way ANOVA, and Bonferroni’s post-test was used to compare data (** *p* < 0.01, *** *p* < 0.001).

We previously showed that SARS-CoV-2 B.1- and B.1.617.2-infected HL-mECs displayed a remodeled phenotype and potent angiogenic activity [20,21]. Due to the lack of infection, HL-mEC dysfunction is likely to be inhibited following cell contact with Omicron BA.5. To this aim, we explored the angiogenic function of HL-mECs following Omicron BA.5 infection. As shown in Figure 5A, HL-mECs infected with Omicron BA.1 developed a consistent network of tube-like structures at 12 h after cell seeding on 48-well plates (4.5 × 10^4^ per well) coated with growth factor-reduced BME, attesting for a potent viral-induced angiogenic activity. At the same time, HL-mECs infected with Omicron BA.5, similarly to mock-infected cells, formed a cellular monolayer on the extracellular matrix, attesting to a lack of angiogenic activity.

Previous data highlighted that angiogenesis observed upon SARS-CoV-2 infection was due to the conditioned microenvironment promoted by infected cells [21]. To demonstrate that the absence of proangiogenic molecules in the secretome of infected cells correlated with a lack of cellular phenotypic changes, we investigated the effect of the SARS-CoV-2-infected HL-mEC secretome on its non-infected counterpart, using a co-culture assay. As shown in Figure 5B, HL-mECs co-cultured for 3 days in the collagen-coated upper insert well of a 0.4 µm pore-size Transwell with Omicron BA.1-infected HL-mECs in the lower chamber, acquired the ability to form a consistent network of tube-like structures when detached and seeded for 12 h on the growth factor-reduced BME. On the contrary, HL-mECs co-cultured with the Omicron BA.5-infected ones, as well as mock-infected HL-mECs, were unable to exert angiogenesis, thus demonstrating that Omicron BA.5 does not foster a proangiogenic microenvironment.

## 4. Discussion

COVID-19 patients with greater severities display dyspnea, chronic obstructive pulmonary disease (COPD), ARDS, and multi-organ dysfunction. The main cause of COVID-19 patients’ mortality is a severe lung injury caused by a cytokine storm, intussusceptive angiogenesis, leukocyte and platelet infiltration, and coagulopathy leading to macro and micro arterial and venous thrombosis [38,39]. Both clinical studies and autopsy findings have also shown vascular damage and thrombotic complications in different organs, as seen in myocardial, liver, kidney, and intestinal injury [4,12]. Increasing clinical evidence suggests a key role of ECs in triggering and sustaining these phenomena by promoting inflammatory, angiogenic, and clotting cascades [40,41,42]. Initial dysfunction of the endothelial barrier and vascular leakage have also been considered responsible for an indirect immune-mediated mechanism of endothelial injury [12,37]. In fact, the accumulation of immune cells at the side of endothelial injury triggers their overactivation with the production of inflammatory cytokines, and angiogenic and vasoactive molecules [43,44,45], further contributing to vascular permeability and edema [12,46,47,48,49,50,51]. More recently, signs of persistent endothelial dysfunction were also shown in COVID-19 patients long after recovery from the infection [9]. Therefore, attention to the role of the endothelium in the pathophysiology process not only in severe but also in long COVID-19 has become of the utmost importance.

The first evidence of ECs infection by SARS-CoV-2 has been provided in human lung biopsies by electron microscopy [52,53,54,55]. Meanwhile, other studies reported that ECs are partially [55,56] or totally resistant to SARS-CoV-2 infection [57,58]. We have recently demonstrated, for the first time, that SARS-CoV-2 is able to infect primary ACE2-negative HL-mECs, by using α_v_β_3_ integrin as an alternate receptor to ACE2 [21]. SARS-CoV-2 binding to α_v_β_3_ integrin occurs through a conserved RGD (403–405: Arg-Gly-Asp) motif that is present in the RBD of the viral Spike proteins. Following infection, HL-mECs support an abortive virus replication without the production of infectious particles. However, viral RNA and/or newly synthesized viral protein expression induced HL-mECs to release a plethora of pro-inflammatory and pro-angiogenic molecules in the extracellular microenvironment and to acquire an angiogenic phenotype. Proteome analysis confirmed a dramatic remodeling of SARS-CoV-2-infected HL-mECs, whereas inhibition of Spike RGD/integrins interaction resulted in blocking SARS-CoV-2 infection and in a complete recovery of viral-induced HL-mEC dysfunction [20,21]. More recently, other studies confirmed the capability of SARS-CoV-2 to infect different ECs [59,60,61] and defined the role of vimentin [62] and heparan sulfates [63] in maintaining and increasing the binding affinity of the viral Spike protein to the EC surface, facilitating SARS-CoV-2 entry.

Here we show that the D405N mutation is mainly expressed in the Spike of all the recently appeared SARS-CoV-2 Omicron subvariants. From a phylogenetic analysis of SARS-CoV-2 variants, we identified in April 2021 an Omicron sublineage, Omicron BA.2, in which a D405N mutation is dominant. The switch from RGD to RGN induced a dramatic loss of function by completely inhibiting SARS-CoV-2 infection of ACE2-negative HL-mECs. This evolutionary trajectory was imposed on the newly emerged Omicron subvariants by the hostile microenvironment generated in response to Omicron BA.1 infection [24] and aimed at preserving viral fitness. Therefore, as unexpectedly appeared in the first SARS-CoV-2 pandemic virus, the RGD motif and its integrin-binding activity disappeared during virus evolution, along with the SARS-CoV-2 capability to infect HL-mECs and to promote endothelial dysfunction. This finding highlights this initial constraint as not being more necessary for further virus adaptation to the human host and, at the same time, supports the early key role of integrins as the main receptors for SARS-CoV-2 entry in ACE2-negative cells. The role exerted by integrins in ECs as alternate receptors to ACE2 for SARS-CoV-2 has been further confirmed by others [64,65].

We can not rule out the possibility that the lack of interaction between Spike’s BA.5 protein and the α_v_β_3_ integrin may be due to other unique BA.5 mutations, such as the R408S, which is also close to the RGD motif and might interact with integrins via electrostatic interaction [66]. At the same time, it is possible that BA.5 mutations other than D405N may impair Spike’s affinity for integrins, as demonstrated for ACE2 with the F486V mutation [67]. This aspect needs to be further investigated in the future.

The key role of ECs in SARS-CoV-2 pathogenesis has been definitively proven in vitro and in vivo [68]. Evidence of mutations retrieving the capability of SARS-CoV-2 to infect HL-mECs highlight a new scenario for patients infected with the newly emerged SARS-CoV-2 Omicron subvariants, suggesting that they may display less severe disease manifestations than those observed with previous variants. Quantifying the intrinsic severity of SARS-CoV-2 variants is extremely challenging [69]. Many factors have changed throughout the course of the pandemic that affects COVID-19 outcomes. This includes variations in the vulnerability of those infected [70], implementation of various public health strategies [71], and introduction of new vaccines and therapeutics [72,73,74,75]. Moreover, one must consider that the impact of SARS-CoV-2 on EC dysfunction is likely to differ among COVID-19 patients, making them more or less prone to develop vascular injury and coagulopathy. In addition, other potential drivers of EC dysfunction, such as low-level SARS-CoV-2 replication in tissues, have to be taken into consideration, together with traditional risk factors for endothelial dysfunction and coagulopathy, including concomitant infections [76], dyslipidemia, hypertension, and diabetes when assessing endothelial injury and thrombotic risks in these patients [77]. After having adjusted data for a variety of confounding factors associated with SARS-CoV-2 outcomes, Strasser et al. [78] recently showed that the Omicron BA.2 subvariant is intrinsically less severe than previous Delta and Omicron BA.1 variants in terms of mortality, risk of hospitalization, intensive care unit admission, and invasive ventilation. The reduced pathogenicity of Omicron can be explained by its less efficient replication compared with prior SARS-CoV-2 lineages in the lung parenchyma, encompassing the alveolar epithelium, in contrast to Omicron’s faster replication in the bronchi [79,80]. To date, different biomarkers are available as both predictors of endothelial dysfunction and injury [81] and of disease progression [82]. However, although preliminary data seem to support the hypothesis that the observed loss-of-function may define the emerging Omicron subvariants as “mild”, clinical and laboratory data need time to be tested with a deeper, scientific dwelling into the facts. Long-term studies with clinical endpoints that include careful measurement of different available markers of inflammation, endothelial injury, and coagulation activity [40,82] will enable clinicians to interpret research data and place them into perspective.

## Figures and Tables

**Table 1:** Analysis of the total number and percentage of isolates carrying different mutations in the Spike RGD motif from Pre-Omicron and Omicron BA.1, BA.2, BA.4, and BA.5 lineages.

		Pre-Omicron		BA.1		BA.2		BA.4		BA.5	
	**All isolates number**	5,280,254		422,746		1,147,103		34,032		26,492	
	**Mutated isolates**	Number	%	Number	%	Number	%	Number	%	Number	%
**R (403)**	**K**	425	0.0080	12	0.0028	9	0.0008	0	0	0	0
	**I**	21	0.0004	0	0	0	0	0	0	0	0
	**S**	14	0.0003	0	0	5	0.0004	0	0	0	0
	**T**	21	0.0004	0	0	0	0	0	0	0	0
	**L**	1	0.0000	0	0	1	0.0001	0	0	0	0
	**G**	15	0.0003	0	0	1	0.0001	0	0	0	0
	**del**	0	0	1	0.0002	3	0.0003	0	0	0	0
	**P**	1	0.0000	0	0	0	0	0	0	0	0
	**W**	1	0.0000	0	0	0	0	0	0	0	0
	**D**	8	0.0002	0	0	0	0	0	0	0	0
		**Total mutated isolates number and percentage**									
		507	0.0096	13	0.0031	19	0.0017	0	0	0	0
**G (404)**	**S**	24	0.0005	0	0	0	0	0	0	0	0
	**A**	13	0.0002	0	0	1	0.0001	0	0	0	0
	**I**	0	0	0	0	1	0.0001	0	0	0	0
	**D**	10	0.0002	1	0.0002	3	0.0003	0	0	0	0
	**C**	67	0.0013	1	0.0002	0	0	0	0	0	0
	**Y**	0	0	2	0.0005	0	0	0	0	0	0
	**V**	10	0.0002	1	0.0002	0	0	0	0	0	0
	**R**	8	0.0002	0	0	0	0	0	0	0	0
	**H**	1	0.0000	0	0	0	0	0	0	0	0
	**K**	1	0.0000	0	0	0	0	0	0	0	0
	**F**	10	0.0002	0	0	0	0	0	0	0	0
		**Total mutated isolates number and percentage**									
		144	0.0027	5	0.0012	5	0.0004	0	0	0	0
**D (405)**	**N**	86	0.0016	827	0.1956	1,114,008	97.1149	33,434	98.2428	26,174	98.7996
	**Y**	180	0.0034	0	0	0	0	0	0	0	0
	**G**	100	0.0019	0	0	0	0	0	0	0	0
	**H**	15	0.0003	1	0.0002	0	0	0	0	0	0
	**E**	34	0.0006	0	0	0	0	0	0	0	0
	**Q**	1	0.0000	0	0	0	0	0	0	0	0
	**M**	0	0	0	0	9	0.0008	0	0	0	0
	**S**	6	0.0001	0	0	3	0.0003	0	0	0	0
	**B**	18	0.0003	336	0.0795	1174	0.1023	4	0.0118	2	0.0075
	**del**	6	0.0001	0	0	4	0.0003	0	0	0	0
	**A**	16	0.0003	0	0	0	0	0	0	0	0
	**C**	1	0.0000	0	0	0	0	0	0	0	0
	**V**	8	0.0002	0	0	0	0	0	0	0	0
		**Total mutated isolates number and percentage**									
		471	0.0089	1164	0.2753	1,115,198	97.2186	33,438	98.2546	26,176	98.8072

## Data Availability

Genomic data for SARS-CoV-2 Omicron BA.1 (accession number: EPI_ISL_15700833), and SARS-CoV-2 Omicron BA.5 (accession number: EPI_ISL_15082179) are available at Global initiative on sharing all influenza data (GISAID). Genomes analyzed in the present study were taken from the GSAID database and are available at https://gisaid.org/ (accessed on 30 November 2022).
